# Avocado Seed Starch-Based Films Reinforced with Starch Nanocrystals

**DOI:** 10.3390/polym16202868

**Published:** 2024-10-10

**Authors:** Pedro Francisco Muñoz-Gimena, Alejandro Aragón-Gutiérrez, Enrique Blázquez-Blázquez, Marina Patricia Arrieta, Gema Rodríguez, Laura Peponi, Daniel López

**Affiliations:** 1 Instituto de Ciencia y Tecnología de Polímeros (ICTP-CSIC), C/Juan de la Cierva 3, 28006 Madrid, Spain; pfmunoz@ictp.csic.es (P.F.M.-G.); enrique.blazquez@ictp.csic.es (E.B.-B.); gema@ictp.csic.es (G.R.); 2 Grupo de Tecnología de Materiales y Envases, Instituto Tecnológico del Embalaje, Transporte y Logística, ITENE, Unidad Asociada Al CSIC, C/Albert Einstein 1, Paterna, 46980 Valencia, Spain; alejandro.aragon@itene.com; 3 Departamento de Ingeniería Química Industrial y del Medio Ambiente, Escuela Técnica Superior de Ingenieros Industriales, Universidad Politécnica de Madrid (ETSII-UPM), C/José Gutiérrez Abascal 2, 28006 Madrid, Spain; m.arrieta@upm.es

**Keywords:** starch, nanocrystals, avocado, seed, antioxidant, biobased films, active packaging

## Abstract

Biopolymers derived from biomass can provide the advantages of both biodegradability and functional qualities from a circular economy point of view, where waste is transformed into raw material. In particular, avocado seeds can be considered an interesting residue for biobased packaging applications due to their high starch content. In this work, avocado seed starch (ASS)-based films containing different glycerol concentrations were prepared by solvent casting. Films were also reinforced with starch nanocrystals (SNCs) obtained through the acid hydrolysis of ASS. The characterization of the extracted starch and starch nanocrystals by scanning electron microscopy, X-ray diffraction, and thermogravimetric analysis has been reported. Adding 1% of SNCs increased elastic modulus by 112% and decreased water vapor permeability by 30% with respect to neat matrix. Interestingly, the bioactive compounds from the avocado seed provided the films with high antioxidant capacity. Moreover, considering the long time required for traditional plastic packaging to degrade, all of the ASS-based films disintegrated within 48 h under lab-scale composting conditions. The results of this work support the valorization of food waste byproducts and the development of reinforced biodegradable materials for potential use as active food packaging.

## 1. Introduction

Plastics are synthetic polymeric materials derived from fossil hydrocarbons. These lightweight, strong, durable, and low-cost materials have become an integral part of everyday life. However, the durability that makes them so appealing becomes a problem as they are highly resistant to degradation in open environments. In this respect, biopolymers are a promising solution as they are obtained from renewable resources and, if disposed of properly, reduce the environmental impact compared to synthetic polymers. Their use can therefore contribute to sustainable development [1]. Of the over 400 million tons of plastic currently produced annually, biopolymers represent approximately 0.5%, with production capacity set to increase from around 2.18 million tons in 2023 to approximately 7.43 million tons in 2028 [2].

The food industry produces large volumes of waste, which results in potential disposal and severe pollution problems. These wastes are also rich in carbon content and can be attractive renewable substrates for the production of added-value products [3]. Avocado (*Persea americana*) is a fruit belonging to the *Lauraceae* family, native to Mexico and Latin America, with Mexico currently being the largest producer worldwide [4]. Avocado peel and seeds are commonly discarded as waste during the processing of avocado pulp. An estimated 520,000–720,000 metric tons of avocado seeds are produced annually worldwide, accounting for 13–18% of the fruit produced annually, estimated to be more than 4 million metric tons [5]. In general, avocado seeds are composed of water (51–58%), starch (27.5–29.8%), sugars (2.2–3.5%), proteins (2.3–2.4%), ash (1.2–1.3%), and crude fiber (3.6–4.1%) [6] and several bioactive compounds such as β-sitosterol and octadecenoic acid. The valorization of these seeds can be performed by the extraction of starch, protein, and phytochemicals, which can be utilized in different industrial applications, including biomaterials, regenerated fibers, pharmaceutical and cosmetic products, animal feed, and biofertilizers [5].

Over recent decades, starch has been actively researched as a potential biopolymer in food packaging applications. Several associated advantages include abundance, biocompatibility, non-toxicity, low cost, biodegradability, and air stability [7]. Native starch granules, upon their botanical source, exhibit a broad range of sizes, size distributions, forms, starch extraction conditions, and chemical composition [8]. Moreover, starch sources can influence films’ properties, enhancing their conceivable applications. However, the brittleness and crystallinity of native starch films greatly limit their application in food packaging [9]. This can be overcome using plasticizers such as glycerol, sorbitol, and urea, frequently incorporated into starch-based bioplastics [10]. To create thermoplastic starch (TPS), the molecular structure should become disordered and the granules gelatinized. During the thermoplastic starch production process, water contained in starch and the addition of plasticizers decreases the internal starch hydrogen bonding, increasing the flexibility of the films [11]. Moreover, plasticizers lower the glass transition temperature and can effectively restrain starch retrogradation, resulting in better packaging qualities [12]. TPS can be achieved via (i) casting from an aqueous system after starch gelatinization, (ii) thermocompression directly from a starch/plasticizer premixture, and (iii) extrusion, i.e., mixing and shear force action at high temperatures [13]. While the latter methods are especially preferred when processing large quantities of material, like on an industrial scale, solvent casting offers a simple and cost-effective method for film preparation, making it more suitable for small laboratory-scale production.

However, starch exhibits some limitations as a stand-alone film material, such as inferior barrier, thermal, and mechanical properties compared to other commercial polymeric counterparts [14]. Common strategies used to enhance starch-based film performance and processability include creating polymeric blends, coating with high-barrier materials, employing multilayered films containing high-barrier films, chemically modifying natural biopolymers, or adding fillers to form composites [15]. In this context, the addition of mineral or organic fillers can be used to create novel, low-cost, biodegradable polymers with reinforced properties. Starch nanocrystals (SNCs) constitute crystalline fragments produced through the disintegration of the amorphous sections in starch granules. The crystalline lamellae, which are more resistant to hydrolysis, can be subjected to extended-time hydrolysis resulting in nanocrystals with dimensions in the nanometer scale (<100 nm) in at least one dimension [16]. Therefore, SNCs can be obtained through the acid hydrolysis (typically using sulfuric acid) of native starch granules by strictly monitoring the temperature, acid and starch concentrations, hydrolysis duration, and stirring speed [17].

Oxidation is one of the main degradative processes that lowers food quality. Packaging with a regulated low-oxygen atmosphere or adding antioxidants to food products are two popular methods for reducing oxidation [18]. Active packaging offers an alternative to these traditional approaches, as it allows the packaging material itself to release antioxidants into the foodstuff. These additives thereby extend shelf life, improve product safety, and can perform additional functions, such as flavor enhancement and gas scavenging [19]. For example, essential oils have a high popularity in film production as the migration of their active compounds can provide improved barrier and optical properties and antioxidant and antibacterial activities [20]. On this note, avocado seeds have been found to exhibit high antioxidant activity attributed to the high content of phenolic compounds and ascorbic acid [21].

Current research on starch-based film production has shifted from traditional starches (i.e., potato, maize, and rice) to non-conventional starch sources such as avocado seeds. Although the use of starch from traditional sources is well established, starch obtained from avocado seeds has two important advantages, which are intended to be highlighted in this study: on the one hand, the use of a waste product from the food industry that is constantly growing; on the other hand, and more importantly, the composition of the avocado seeds allows the incorporation of intrinsic antioxidant and antimicrobial compounds into the developed films.

The main aim of this study was to produce plasticized starch-based films using avocado seeds as the starch source and to reinforce them with starch nanocrystals. Before processing the films, a preliminary study was carried out to determine the properties of ASS and SNCs. While increasing the plasticizer concentration increases film flexibility, elastic modulus, and elongation, it also decreases the film’s barrier properties. Therefore, ASS was combined with varying glycerol concentrations to compare the properties of the films in search of a compromise between all properties. As reinforcing agents, SNCs obtained through acid hydrolysis were added in different amounts to the preferred TPS formulation (Gly35) to reduce film permeability. Finally, the mechanical, morphological, thermal, antioxidant, barrier, and biodegradation properties of all of the developed films were studied and evaluated to establish the best composition.

## 2. Materials and Methods

### 2.1. Reagents and Materials

The avocado seeds obtained from avocado (*Persea americana Miller*, cv. Hass) fruits were purchased in a local grocery store in Madrid, Spain. Glycerol (purity ≥ 99%, CAS 56-81-5), sodium bisulfite (CAS 7631-90-5) and iodine (CAS 7553-56-2) were acquired from Sigma-Aldrich (St. Louis, MO, USA). Potassium iodide was purchased from Quimicen SA (Madrid, Spain), 2,2-difenil-1-picrylhydrazyl (DPPH), and sulfuric acid (CAS 76664-93-9) was obtained from Scharlau (Barcelona, Spain).

### 2.2. Avocado Starch Production

Starch was extracted from avocado (*Persea americana Miller*, cv. Hass) seeds, based on a modified procedure described by Chel-Guerrero et al. [22]. Briefly, seeds were removed from fresh avocados and washed with water. Then, 100 g of seeds were peeled, cut into small pieces, and introduced in a sodium bisulfite solution (NaHSO_3_) (1500 ppm) 1:5 (*v*/*w*) to prevent oxidation. The seeds were milled in NaHSO_3_ solution using an IKA Multidrive blender (Staufen, Germany) at 10,000 rpm for 1 min followed by 3 min at 3000 rpm (10 s running and 5 s rest). The resultant suspension was filtered using a 50 µm mesh sieve. The starch was washed with distilled water and dried using vacuum filtration. The resulting avocado seed starch (ASS) was stored in a sealed container at room temperature.

### 2.3. Avocado Starch Nanocrystal Elaboration

Avocado starch nanocrystals were produced by the acid hydrolysis of the previously prepared ASS, using a modified version of the procedure described by Angellier et al. [23]. The starch (10 g) was dispersed in 200 mL of 3.16 M H_2_SO_4_ solution inside a 250 mL glass tank reactor equipped with a magnetic stirrer. The reaction was left stirring for 5 days at 40 °C and 250 rpm, to hydrolyze the amorphous fraction. The reactor temperature was kept lower than the gelatinization temperature and argon was bubbled into the suspension to reduce oxidation. After 5 days, the suspension was diluted with 2 L of distilled water, stirred, and left to decant. The precipitate was re-suspended in distilled water and centrifuged for 10 min at 10,000 rpm. The washing process was repeated until it reached a neutral pH (5.5). The nanocrystals were freeze-dried and stored in a dry chamber at room temperature.

### 2.4. Avocado-Starch-Based Film Formation

ASS-based films were all prepared from a film-forming solution of 1% wt. avocado starch powder (0.3 g starch in 30 mL distillate water). The additives were added in relation to the mass of avocado starch and expressed in parts per hundred resin/rubber (phr). The glycerol content was 0, 15, 25, 35, and 45 phr, as seen in Table 1. The solution was heated in a silicone bath with mechanical stirring for 15 min at 95 °C for complete gelatinization. Then, the films were obtained by solvent casting in a polystyrene Petri dish in an oven at 37 °C for 24 h. SNC concentrations (1, 3, and 5 phr) were added to the Gly35 solution after the mixture had cooled down to 40 °C. The SNCs were dispersed using an Ultra-Turrax T25 (IKA, Staufen, Germany) at 8000 rpm for 5 min before casting.

### 2.5. Avocado Starch and Starch Nanocrystal Characterization

The chemical structure of ASS and SNC was studied by Fourier-transform infrared (FTIR) spectroscopy, using a Spectrum One FTIR spectrometer (Perkin Elmer instruments, Madrid, Spain). The samples were analyzed at room temperature in the spectral range of 400–4000 cm^−1^ at a resolution of 4 cm^−1^.

The percentage of amylose in the starch was determined following the total amylose content method [24]. Briefly, the starch was placed in a cellulose extraction thimble inside a Soxhlet extractor. Lipids were removed with 200 mL of a 75% (*v*/*v*) n-propanol solution in water heated at 110 °C for 24 h. The thimble was air-dried for 12 h before removing the lipid-free starch. The starch was placed in an oven at 37 °C for 24 h. Triplets of the dried starch sample (20 mg) were dissolved in 8 mL 90% DMSO–water solution and heated at 85 °C for 15 min with intermittent mixing. The samples were diluted to 25 mL with deionized water, of which 1 mL was diluted in 50 mL dilution with 5 mL of iodine solution. Similarly, 20 mg mixtures of pure amylose and amylopectin standards (0%, 10%, 20%, 40%, 50%, 60%, 80%, 90%, and 100% amylose) were prepared. The absorbance of samples and standards were measured in a UV-Vis Varian Cary 1E UV-Vis spectrophotometer (Madrid, Spain) at 600 nm against a reagent blank as a reference.

The crystallographic structure was analyzed using a Bruker D8 Advance instrument (Madrid, Spain) with a CuK as source (0.154 nm) and a Detector Vantec1. The operating conditions were X-ray line (λ = 1.5406 Å), voltage 40 kV, and current 40 mA. The samples were loaded on an aluminum plate and X-ray diffractions were recorded from 0° to 60° for 2θ with a scanning rate of 0.02°/min.

The thermal stability of the starch, starch-based films, and nanocrystals were studied using a TA Instruments TGA Q500 analyzer (Waters, Madrid, Spain). Samples (~10 mg) were heated from 30 to 800 °C at a heating rate of 10 °C min^−1^ under a nitrogen atmosphere. Previously, the samples were introduced into a desiccator with phosphorus pentoxide to minimize the water content. The maximum degradation temperature (Tmax) was calculated from the first derivative of the TGA curves. Dynamic DSC measurements were performed in a Mettler Toledo DSC822e instrument (Barcelona, Spain). The starch gelatinization process was studied under a nitrogen atmosphere: samples of around 2 mg were sealed in aluminum pans in an excess of water and heated from 25 °C to 100 °C at a heating rate of 10 °C/min.

### 2.6. Film Characterization

Film thickness was determined using a digital micrometer (0.001 mm, Hangzhou United Bridge Tools Co., Ltd., Hangzhou, China). For each formulation, ten random points were measured on three different films. The color properties of the films were evaluated by measuring the color coordinates in the CIELab color space: L* (lightness), a* (red-green) and b* (yellow-blue) employing a KONICA CM-2500d (Konica Minolta Sensing Americas, Inc.,Wayne, NJ, USA). The instrument was calibrated using a white and black standard tile and the measurements were taken at random locations over the surface of the TPS-based films. The results are expressed as the average values of at least five measurements.

The morphology of cryo-fractured cross-sections of films was studied using a SEM (PHILIPS XL30 Scanning Electron Microscope, FEI Philips, Hillsboro, OR, USA). All of the samples were previously gold-coated (≈5 nm thickness) in a Polaron SC7640 Auto/Manual Sputter. SEM images were acquired with an accelerating voltage of 25.0 kV and a working distance of 20 mm.

The mechanical properties of the films were studied using an Instron Universal Machine (Barcelona, Spain) at a strain rate of 10 mm min^−1^. Tensile test measurements were performed on dog bone specimens with a width of 2 mm, leaving a 20 mm separation between the clamps. The strips were preconditioned at 40% relative humidity (RH) for 24 h inside a sealed container at room temperature (25 ± 1 °C). The Young Modulus, the elongation at break, and the tensile strength were obtained.

The water permeability of the films was measured by determining the water vapor transmission rate (WVTR) by gravimetry. The films were placed in permeability cups exposed to an area (A) of 10 cm^2^. The test cups were filled with 2 g of silica gel as a desiccant to produce a 0% RH below the film. The permeability cups were kept in a desiccator with a saturated KNO_3_ solution at 23  ±  1 °C and under an RH higher than 75%. Three samples of each formulation film were weighed each hour for 8 h. WVTR (g days^−1^ cm^−2^) was calculated using Equation (1), where m_t_ is the mass of the cup at time t, m_0_ is the initial mass of the cup, and A is the exposed area of the films.

(1) $$WVTR=\frac{x\left(m_{t}-m_{0}\right)\;*\;240}{A\;*\;t}$$

The water vapor permeability (WVP) was calculated according to Equation (2), where S is the saturation vapor pressure of water at 23 °C, R1 and R2 are the relative humidity in the desiccator and inside the cup, respectively, and ε is the thickness of the film.

(2) $$WVP=\frac{WVTR\;*\;\varepsilon }{S\;*\;\left(R1-R2\right)}$$

The oxygen transmission rates of the films were determined using an OX-TRAN Model 2/22 H OTR Analyzer Mocon (Lippke, Neuwied, Germany). Tests were carried out at 23 ± 1 °C and 0% relative humidity following the ASTM D3985 standard [25].

For fatty food products aimed to be stored in ambient conditions (temperatures between 20 °C and 40 °C for not more than 30 days), the legislation establishes that food packaging materials must be tested at contact temperature in the worst foreseeable use of 40 °C for 10 days. Therefore, double-sided total-immersion-specific migration tests were conducted via the total immersion of two of 15 × 15 mm film samples in a glass vial containing 7.5 mL of fatty food simulant (Simulant D1 = ethanol 50% *v*/*v*) at 40 °C for 10 days [26]. Simulant D1 was run simultaneously with the migration test and used as a control, and all samples were performed in triplicate. After 10 days, the film samples were removed and the radical scavenging activity (RSA) of the food simulant was determined to measure the antioxidant capacity of starch-based films by the reduction of the absorbance of a 2,2-difenil-1-picrylhydrazyl radical (2mM DPPH in ethanolic solution) at 526 nm using a UV-Vis Varian Cary 1E UV-Vis spectrophotometer. The percentage of the radical scavenging activity (RSA) was determined using Equation (3).

(3) $$RSA\left(\%\right)=\frac{A_{control}-A_{sample}}{A_{control}}\times 100\%$$

where *A_control_* is the absorbance of DPPH radical in simulant D1 and *A_sample_* the absorbance of DPPH solution after 15 min in contact with each food simulant sample after 10 contact days with the starch-based films.

The analytical determination of antioxidants was carried out using a Hewlett Packard 6890 HRGC gas chromatograph equipped with an Agilent Technologies mass spectrometry detector model 5973 (GC-MS) (Santa Clara, CA, USA). The separation of the compounds was performed on a DB5-HT capillary column (15 m × 250 μm and 0.1 μm). The carrier gas used was helium with a flow rate of 1 mL/min. The electronic impact (70 eV) was the type of ionization selected for the mass spectrometer. The different samples were extracted taking 50 mg and adding dichloromethane at 40 °C for 24 h. The solvent was evaporated until dryness and the extracts were analyzed by GC-MS. The identification of compounds was carried out by matching their mass spectra vs. the NIST20 (US National Institute of Standards and Technology, Gaithersburg, MD, USA) commercial library, with a match above 900.

The films were disintegrated under industrial composting conditions at a laboratory scale following ISO 20200 standards [27]. The films were cut into 15 × 15 mm samples, placed in mosquito nets, and buried in a perforated plastic box with prepared compost soil. The mosquito net allowed direct contact with compost soil and facilitated the removal of degraded film. The compost soil consisted of a mixture containing approximately 40% sawdust, 30% rabbit food, 10% compost, 10% corn starch, 5% sugar, 4% corn oil, and 1% urea. Deionized water was added up to 55 wt.% of the total content. The boxes were kept under aerobic conditions at 58 °C. The samples were unburied after 1, 3, 6, 12, 24, 36, and 48 h, and dried in an oven at 37 °C for 24 h. Photographs of unburied samples were taken for visual comparison.

## 3. Results and Discussion

### 3.1. Starch Granule and Nanocrystal Characterization

The starch extracted from the avocado seeds (ASS) exhibited a light brown color. The starch nanocrystals (SNCs) showed a slightly darker brown color compared to the ASS granules. ASS extraction yielded about 13.1%, which is similar to the 20.1% reported using a similar sodium bisulfite solution [22] or the 16.5% (wet basis) obtained through a combined mechanical and ultrasonic extraction method [28]. The iodine method established that the amylose content of the extracted avocado starch was 31.4%, which is similar to the 29.7 ± 2.4% [29] reported for ASS from an unspecified avocado variety. In solvent casting, when comparing films obtained in the same conditions, starch matrices with higher amylose percentages are thicker and, thus, more opaque, while amylopectin content favors obtaining less heterogeneous and denser films and, thus, thinner films [30]. High amylose starches have a higher viscosity, making processing techniques challenging, resulting in TPS with higher stiffness, therefore increasing elastic modulus and tensile strength and decreasing elongation [31].

The FTIR spectra for ASS and SNCs and a description of their characteristic bands can be seen in Supplementary Figure S1. The similarity in the spectra suggests that the chemical bonds of the SNCs remained mostly unchanged after the acid hydrolysis. The absorption peak intensity of SNCs increased in the 3000–3600 cm^−1^ and 1635 cm^−1^ band compared to ASS; this is due to the increasing hydroxyl groups in the SNCs by acid hydrolysis. Similarly, the band at 2930 cm^−1^ was lower in SNCs, which is ascribed to decreased amylose content, which was also expected, since amylopectin is predominant in the crystalline fraction [32]. The small variations in the 1050–990 cm^−1^ region could be associated with the presence of more ordered structures and the disappearance of the amorphous fraction, respectively [33].

A thermogravimetric analysis of the ASS and SNCs was carried out to compare their thermal stability. As seen in Supplementary Figure S2, the TGyadaA curves show three stages. The initial weight loss (60–120 °C) corresponds to the water evaporation and low-molecular-weight compounds (approx. 7–8% of weight). The main weight loss (250–330 °C) refers to the thermal degradation of carbohydrates including amylose and amylopectin, as well as the eradication of lipid complexes, through an irreversible reaction, producing water, carbon monoxide, carbon dioxide, acetaldehyde, furan, and 2-methyl furan. The last stage (above 330 °C) is related to the carbonization decomposition of raw starch, which ends with the formation of carbon black.

The SNCs exhibited lower water content (3–4% of the weight) compared to the ASS. This reduction can be associated with the higher crystallinity, therefore being structurally more compact and organized, and the presence of sulfate groups that reduce the exposure of hydroxyl groups on the surface, reducing the water content, as reported by Velásques et al. [34]. Additionally, the thermal decomposition of SNCs began earlier than the ASS granules despite the hydrolysis treatment. This is consistent with relevant literature findings in which the depolymerization reaction of SNCs is catalyzed faster by the presence of sulfate groups produced during hydrolysis on the surface of SNCs [35]. Interestingly, LeCorre et al. [36] compared different starches and their SNCs, and suggested that larger SNCs have higher degradation temperatures, closer to the initial starch thermal behavior, as groups with less sulfate can catalyze the degradation reaction. Moreover, at 200–300 °C, the sulfonic acid groups are thermally decomposed and the depolymerization rate slows down, with no influence on the final weight. The earlier thermal decomposition of SNCs does not affect their application in solvent-casted film. However, the preparation of nanocomposite films using other thermal methods can result in the melting of SNCs, especially if water is present [37].

Starch suffers gelatinization in excess water. The DSC analysis showed a gelatinization curve of avocado starch, with a maximum peak centered at 71 °C. The recorded initial gelatinization temperature was 63 °C and the final gelatinization temperature was 82 °C (Supplementary Figure S3), in agreement with other literature results. For example, Lacerda et al. [38] reported a gelatinization range of 73.77 to 81.27 °C for an unspecified variety of avocado starch.

**Figure 1:**
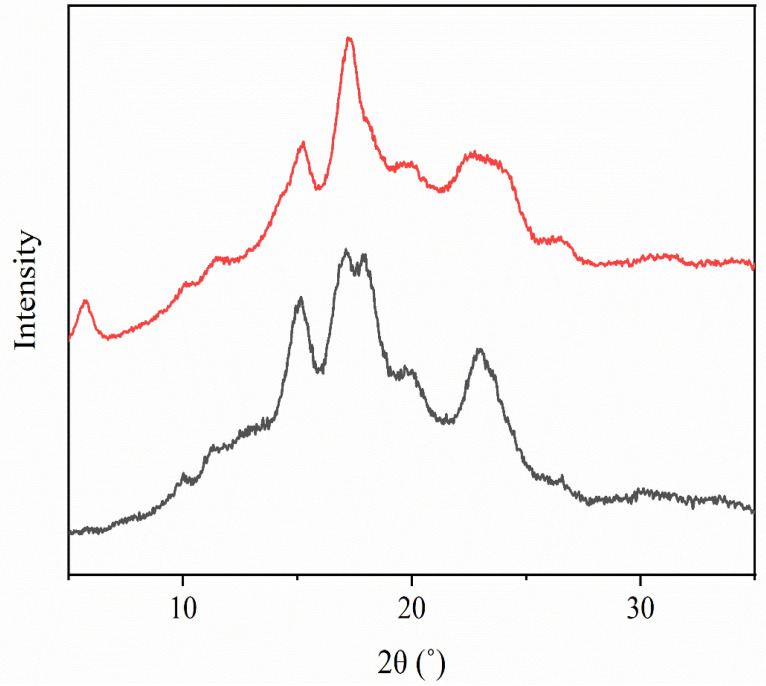
X-ray diffraction patterns of ASS (top) and SNCs (bottom).

Wide-angle X-ray diffraction (XDR) was carried out on the ASS granules and SNCs, as shown in Figure 1. The XDR patterns for ASS showed a strong peak at 17.1° (2θ) and other peaks at 2θ 15.2°, 19.6°, and 22.4°, exhibiting a clear B-type pattern. This result matches the B-type patterns commonly described for Hass ASS, as described by De Dios-Ávila et al. [39]. SNCs presented changes in the crystallinity pattern after hydrolysis, mainly the strong peak at 17.1° unfolding into a double peak. Moreover, the SNCs showed an increased reflection peak intensity compared to the ASS. This is expected as a result of the selective hydrolysis of the amorphous regions of starch by the sulfuric acid, subsequently forming a more defined crystalline region.

**Figure 2:**
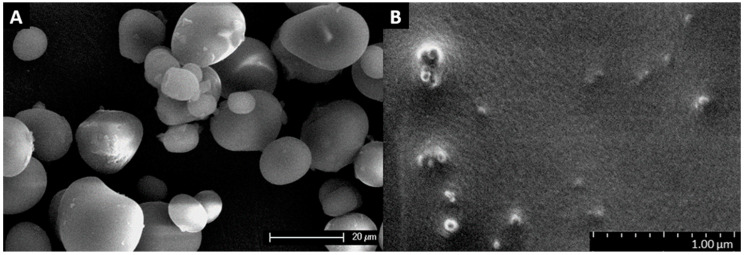
(**A**) SEM micrograph of avocado seed starch (ASS) granules. (**B**) FE-SEM micrograph of starch nanocrystals (SNCs).

The morphology of the ASS and SNCs was studied using a scanning electron microscope (SEM and FE-SEM). As seen in the SEM micrographs in Figure 2A, the ASS granules exhibited different forms including rounded, elliptical, and irregular shapes. Moreover, the granules exhibited a smooth surface with varying granular sizes, including small rounded 3–7 μm granules and larger elliptical and irregularly shaped granules, with lengths between 15 and 30 μm and widths of 5–20 μm. This matches the description reported by Esquivel-Fajardo et al. [28], whose ASS granules exhibited lenticular, spherical, and irregular morphologies, with a 5–30 µm size distribution, a mean length of 18.6 ± 5.2 μm, and a mean width of 10.9 ± 3.1 μm.

The FE-SEM observation of SNCs showed crystals with differing dimensions and shapes, as seen in Figure 2B. Most SNCs exhibited a round shape with diameters smaller than 100 nm. This description would fit a study by LeCorre et al. [40], where they explained how B-type starches (e.g., high amylose maize, potato) produced round-shaped particles after acid hydrolysis. Moreover, the SNCs presented a strong tendency to self-aggregate, which could be explained by surface hydroxyl groups promoting hydrogen bonding interactions, as described by Bel Haaj et al. [41].

### 3.2. Film Characterization

**Figure 3:**
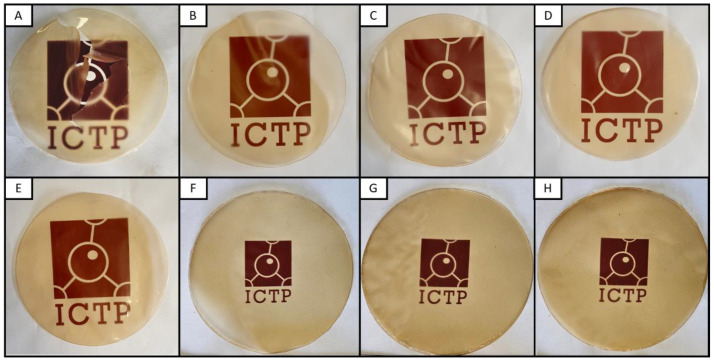
Visual aspect of avocado-starch-based films: (**A**) Gly0, (**B**) Gly15, (**C**) Gly25, (**D**) Gly35, (**E**) Gly45, (**F**) Gly35-1SNC, (**G**) Gly35-3SNC, (**H**) Gly35-5SNC.

Visual aspects of the avocado seed starch (ASS)-based films are presented in Figure 3. On the macroscopic scale, all of the films obtained were homogeneous, continuous, and odorless and exhibited a light orange/brown color. Glycerol and SNCs had no significant effect on film transparency and color, as seen in Table 2. The lightness (L*) of all of the examined films was higher than the 73.02 ± 0.48 reported by Jímenez et al. [42] for their plasticized ASS films. Moreover, the same films presented higher red (a*: 12.47) and yellow (b*: 31.58) values. All films exhibited a* y b* coordinate values closer to light white, similar to those reported for banana starch [(L*) 81.33, (a*) 4.29, and (b*) 10.07] [43]. Despite the ultrasonic dispersion of SNCs before casting, small macroscale agglomerations were visible, mainly in the highest concentration (Gly35-5SNC). The films were easy to handle, except Gly0 and, to some extent, Gly15, which presented a fragile nature. The unplasticized film (Gly0) formed very brittle and cracked films, limiting possibilities to measure mechanical and barrier properties.

Film thickness was significantly influenced by glycerol and SNC content, as seen in Table 2. Higher glycerol concentrations lead to thicker films. This is expected, as the matrix becomes less dense and the mobility of starch chains is facilitated with the increase in plasticizer [44]. Zaharia et al. [45] reported that glycerol concentration increased potato starch film thickness and moisture content while decreasing density. On the other hand, Gly35-1SNC films had significantly lower thicknesses compared to Gly35. Additionally, Gly35-3SNC and Gly35-5SNC displayed small increases in thickness compared to Gly35 despite the increment in total dry mass content. A possible explanation is that SNCs dispersed homogeneously in the starch matrix, making films with higher density. Fan et al. [46] reported similar behavior, where the average thickness of the control starch film was 160 µm compared to the 120 µm of the 1% SNC film.

**Figure 4:**
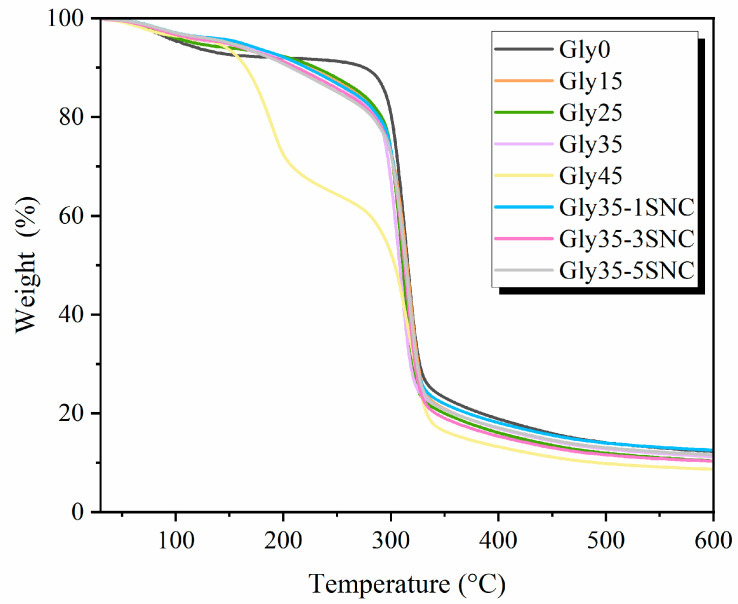
Thermogravimetric analysis of avocado seed starch (ASS)-based films with different glycerol and starch nanocrystal (SNC) content.

The effect of glycerol and SNCs on the thermal properties of the ASS films was studied by thermogravimetric analysis, as seen in Figure 4. The initial stages of film thermal degradation originated at the 60–120 °C interval. This weight loss can be connected with moisture and the evaporation of loosely connected water and low-molecular-weight molecules in the film. The plasticized films presented lower T_10%_ than the unplasticized films (Gly0), which can be attributed to the reduction in inter- and intra-molecular bonds in the starch matrix as the concentration of glycerol increased. Similarly, the addition of SNCs further reduced T_10%_, indicating a lower water absorption capacity. The strong hydrogen bond between SNCs and ASS probably prevented water absorption in the starch matrix.

The main degradation process (250–330 °C) is linked to the loss of hydroxyl groups and depolymerization of carbon chains. The TGA analysis of the glycerol films showed a constant decreasing trend in Tmax as the plasticizer content increased. The lower thermal stability of glycerol compared to ASS can explain this phenomenon. Interestingly, Gly45 displayed two phases during the main degradation the first at 150–200 °C and a second at 250–320 °C, demonstrating a phase separation between the glycerol and the starch. The addition of SNCs resulted in lower initial decomposition temperatures, as seen in Figure 4, as nanocrystals exhibit lower initial thermal stability after the hydrolysis processing of the native starch granules. However, increasing the SNC concentrations also resulted in higher T_max_ with Gly35 (307.4 °C), Gly35-1SNC, (312.6 °C), Gly35-3SNC (313.5 °C), and Gly35-5SNC (314.2 °C), which evidenced that thermal stability of films was enhanced with the incorporation of SNC. Similarly, Kumari et al. [47] described how the addition of SNC increased the T_50%_ of their mung-bean-starch-based films by up to 7 °C and attributed it to the strong crystalline structure of SNC, which required more energy and time to melt the crystal. All films presented a similar mass residue at 600 °C, around 10% of the remaining mass, matching the previously described TGA pattern for ASS and SNCs.

**Figure 5:**
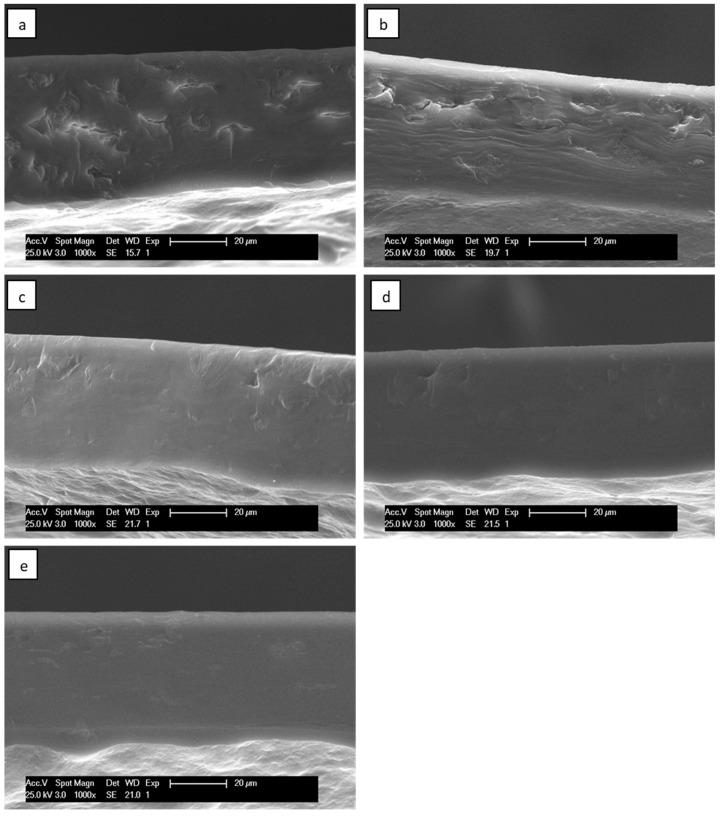
SEM micrographs (1000×) of the cross-section of avocado starch films with varying plasticizer content: (**a**) Gly0, (**b**) Gly15, (**c**) Gly25, (**d**) Gly35, (**e**) Gly45.

The cryo-fractured cross-sections of all of the films were studied by scanning electron microscopy to assess the influence of the addition of glycerol and SNCs on their morphology. Noteworthy differences were spotted between films when comparing different glycerol content, as seen in Figure 5. The unplasticized film (Gly0) presented small cracks on the surface, indicating a fragile nature, which could explain the difficulties in forming a complete film. The addition of glycerol (Gly15) significantly decreased the number and size of the cracks, while an even higher glycerol content (Gly25, Gly35, and Gly45) resulted in smooth surfaces, confirming good gelatinization and the homogeneous mixing of ASS with glycerol. The small scale of glycerol molecules grants enhanced intercalation capabilities between the polymeric chains. The high compatibility permits glycerol to reduce the strong intramolecular attraction in the starch matrix and increase the H-bonding between the plasticizer and starch molecules. The homogeneity of these films, which can be attributed to this good synergy, results in better mechanical properties.

**Figure 6:**
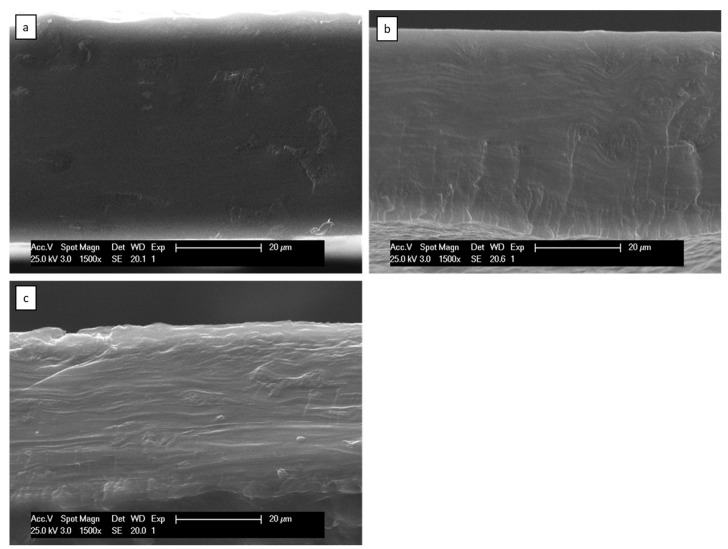
SEM micrographs of the cross-section of SNC-reinforced avocado starch films (1500×): (**a**) Gly35-1SNC, (**b**) Gly35-3SNC, (**c**) Gly35-5SNC.

The influence of SNCs in the morphology was also studied, as seen in Figure 6. The smooth surface of the original Gly35 film can be seen in all SNC formulations. However, small agglomerations of SNCs can be perceived in Gly35-5NC and, to a lesser extent, in Gly35-3SNC. The tendency of SNCs to self-aggregate into microscale agglomerates is a common behavior, independent of the morphology of nanocrystals and botanical origin of the starch [37].

The wide-angle X-ray diffraction patterns of the ASS films are shown in Supplementary Figure S4. The films showed diffraction peaks and a broad amorphous halo, which is the typical behavior of semi-crystalline polymers with a low degree of crystallinity. Diffraction peaks can be identified at 2θ of 12.9°, 17.1°, and 19.9°. Although ASS presented a B-type crystallinity, the films obtained showed a pattern of VH-type crystallinity. This indicates that the crystalline structure of native starch was destroyed during gelatinization, and a semi-crystalline structure was formed from the recrystallization of simple amylose helices (B-type crystallinity), as reported by Palluch et al. [48]. The VH-type crystallinity is induced during the heat treatment, where the interaction between the hydroxyl groups of the starch molecules is replaced by hydrogen bonds formed between the plasticizer and starch [49]. The trends of XDR analysis of the glycerol films showed a constant decrease in peak intensity at 13.0° as the plasticizer content increased. On the other hand, an increase in the peak intensity at 13.0° and 17.0° was observed mainly at Gly35-3SNC and Gly35-5NC. This could be the result of the macroscale SNC agglomerates formed in the nanocomposite at the highest SNC concentration.

The mechanical properties of starch films exhibit high sensitivity to multiple factors including botanical origin and humidity. A high relative humidity can provide a plasticizing effect, as water molecules enhance molecular mobility due to an increase in free volume. The unplasticized films (Gly0) were rigid and brittle; consequentially, no measures for mechanical properties could be taken. The plasticizing effect of glycerol can be seen in Table 3a. Films with increasing glycerol content exhibited a lower elastic modulus and tensile strength, while the elongation at break increased. The elastic modulus values decreased significantly from 1247 MPa to 109 MPa with glycerol incorporation, which translated into lower rigidity and stiffness in the films.

The addition of a small concentration of SNCs (Gly35-1SNC) led to an increase in the elastic modulus and tensile strength, and a decline in elongation at break, as seen in Table 3b. The high specific surface area provided by the SNCs caused stronger filler–matrix interfacial interactions, leading to the higher tensile strength (+112%) of the films reinforced with starch nanoparticles. However, the higher quantity of SNCs in Gly35-3SNC provided a smaller reinforcing effect compared to Gly35-1SNC, while Gly35-5SNC presented values similar to its neat counterpart (Gly35). This is to be expected, as high concentrations of SNCs tended to self-aggregate, decreasing the surface area for interactions with the matrix, resulting in decreases in tensile strength and modulus. Velásquez-Castillo et al. [33] reported that their cassava starch film tensile strength (TS) and Young’s modulus (M) increased (+154% and +135%, respectively) when the quinoa SNC concentration increased from 0 to 5%, and then reduced at 7.5% of QSNC. Similarly, Li et al. [50] reported that higher SNC concentrations had a reduced reinforcing effect on their pea starch films and associated it with the linking of hydroxyl groups in the surface of the SNCs, leading to a micro-phase separation.

Since the main function of food packaging is often to avoid or at least to decrease moisture transfer between the food and the surrounding atmosphere, water vapor permeability (WVP) should be as low as possible. Therefore, the high water permeability of starch films limits their use in the food packaging industry. The WVP values of the ASS films increased with higher glycerol content, as seen in Table 4. Glycerol can easily penetrate the starch film structure, decrease the matrix density, and create active sites where water molecules can be absorbed. Therefore, rising glycerol content forms a hydrophilic film with poor moisture barrier properties, as it increases moisture content, water solubility, and water absorption.

On the contrary, the incorporation of SNCs significantly reduced the WVP of the starch films (Gly35), as seen in Table 4. Gly35-3SNC exhibited a 40% decrease in WVP compared to Gly35. It is well known that adding nanoparticles to composite films forms a tortuous path for water molecules to permeate, significantly decreasing WVP. However, the high amount of hydroxyl groups at the surface of starch nanocrystals can lead to an interaction with water and a consequent increase in WVP. In this work, the reduction in the WVP can be ascribed to the positive interaction between the polymeric matrix and the SNC that avoids the interaction with water. The strong interactions between the nanoparticles and the polymeric matrix enhance film compactness, reducing possible paths for water molecules trying to penetrate the polymeric matrix.

The oxygen transmission rate (OTR) results for the films are shown in Table 4. The brittle nature of non- or slightly plasticized films combined with the small measurement area (2.5 cm^2^) resulted in small cracks that caused the OTR values to be above the upper measurement range of the equipment for the tested area. However, it is commonly recognized that adding plasticizers lowers the oxygen barrier properties of the matrix by increasing free volume. Therefore, OTR values should increase as glycerol content also increases. On the other hand, the addition of SNCs has been reported to lower OTR in films [51]. A decreasing pattern can be seen when comparing Gly35-1SNC and Gly35-3SNC to Gly35. However, for Gly35-5SNC films, a possible agglomeration of SNCs resulted in lower barrier properties. Nevertheless, compared to traditional plastics, the poor oxygen and water vapor barrier properties are still insufficient to serve as high-barrier packaging, greatly limiting the application of current biodegradable polymers. As a result, the current focus of biodegradable food packaging is mainly on dry food products for short shelf lives, or long shelf-life applications that do not require strong barrier properties.

**Figure 7:**
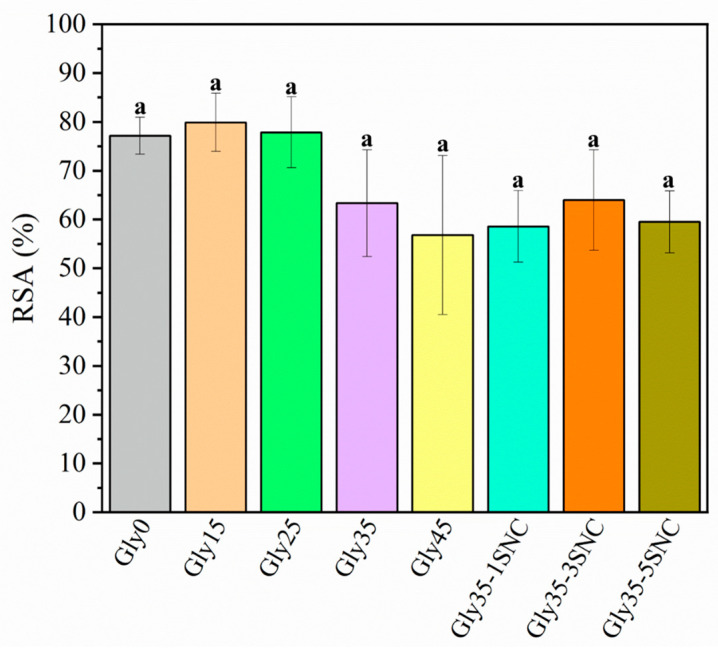
Radical scavenging assay (RSA) effect of glycerol and starch nanocrystal content on avocado starch films. Different letters in the column indicate significant differences according to Tukey’s test (*p* < 0.05). F ratio: 1.208 and *p*-value > F: 0.353 (Values are significant at *p* < 0.05).

Radical scavenging activity (RSA) was measured using the DPPH method after the materials were subjected to specific migration tests. In this technique, when antioxidant substances are present in the sample, the initial purple color of the solution changes to yellow. This color change is triggered by antioxidants donating hydrogens from their phenolic hydroxyl groups to DPPH free radicals, resulting in a more stable compound [52]. All of the films presented antioxidant activity, as seen in Figure 7, since the ASS polymeric matrix is rich in antioxidant compounds.

Although the RSA values were similar for all films, a downward tendency can be seen, meaning that the antioxidant ability decreased as the glycerol content increased. Thus, the lowest RSA% was presented by Gly45 as glycerol does not possess any antioxidant activity, while the highest value was seen for films containing lower amounts of glycerol (Gly0, Gly15, Gly25). Additionally, the antioxidant capacity of the ASS was probably further reduced due to interactions between the starch and glycerol. Similar results were reported by Merino et al. [53], in which pure avocado seed powder presented a higher antioxidant capacity than plasticized film. The presence of SNCs had no apparent effect on the film’s antioxidant capacity, as the values remained comparable to Gly35. Hu et al. [54] developed cassava starch aldehyde functionalized with catechin conjugates reaching around 60% RSA values, similar to those obtained here. However, they required the incorporation of catechin conjugates in high amounts (between 18% and 62%) into the starchy structure. Meanwhile, the materials developed here intrinsically possess antioxidant activity.

Waste materials from the avocado processing industry (peels and seeds) have been proven to show intense antioxidant effects linked to polyphenols, which can vary between avocado varieties [55]. ASS, SNCs, and Gly35 film were analyzed by GC-MS to identify the source of the antioxidant properties of the films. A series of proposed compounds were identified in all three samples, as seen in Table 5. Furan and furanone derivatives isolated from avocados have notable insecticidal, cytotoxic, and antifungal activities [56]. n-Hexadecenoic acid is reported to have antioxidant, anti-inflammatory, hypocholesterolemic, and cancer-prevention properties [57]. Similarly, 9,12-octadecadienoic acid has been linked to anti-inflammatory, antibacterial, hypocholesterolemic, and hepatoprotective activities [57]. On another note, avocado fruits have been reported to be a good source of 4-desmethylsterols, with β-sitosterol representing around 89% of total sterol content [58]. β-sitosterol has anti-inflammatory, antibacterial, and antifungal effects [59].

**Figure 8:**
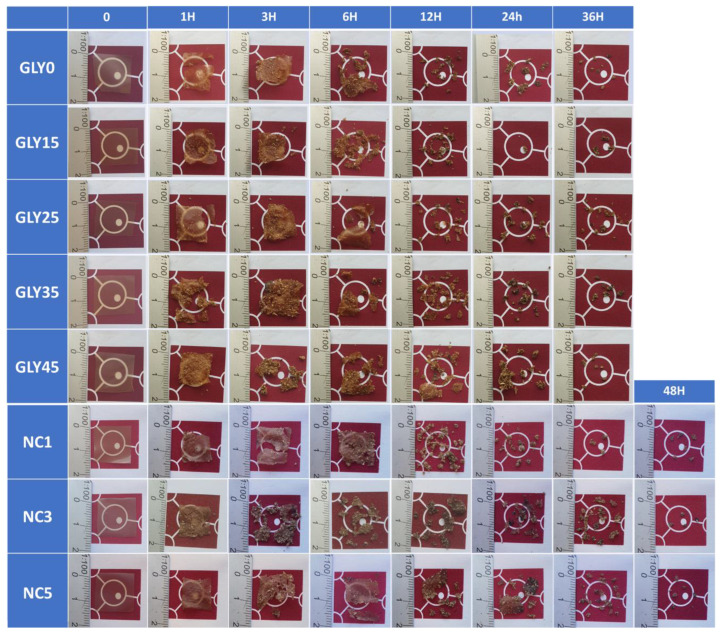
The visual appearance of avocado-starch-based films during disintegration.

The disintegration test reproduces the humidity (about 55%) and heat (58 °C) conditions typical of the thermophilic phase of a composting plant at a lab scale. The degradation rate was studied under visual comparison, as seen in Figure 8. After 12 h, all films presented clear degradation as they had lost their initial appearance, shape, and structural integrity. Moreover, all of the starch films were fully degraded after 48 h. Sessini et al. [60] observed that starch films with a lower amount of glycerol disintegrated faster under lab-scale compost conditions (58 °C), explaining how the chain mobility of plasticized films allowed the water to infiltrate through the polymer matrix, leading to earlier hydrolysis. A lower degradation rate was observed in the nanocomposite films containing SNCs. These results could be related to the nucleation effect of SNCs on the films, since the ordered structures in the crystalline fractions decrease the action of microorganisms [61].

## 4. Conclusions

Starch was successfully extracted from avocado seed and characterized. Starch-based films with intrinsic antioxidant properties were obtained through the addition of glycerol and starch nanocrystals. Increasing the glycerol content reduced the brittleness and tensile strength of the films, but lowered the barrier properties. The Gly35 film was found to be a good compromise between these properties and was therefore selected to incorporate the starch nanocrystals (SNCs) prepared via acid hydrolysis. The addition of SNCs created a tortuous path that lowered water vapor and oxygen permeability. The reinforcing effect was especially effective at lower SNC concentrations, as higher concentrations led to SNC agglomeration that hindered the interactions between the matrix and SNCs. Considering all of the studied properties comprehensively, Gly35-1SNC presented the best balance of properties: high tensile strength and modulus, and moderate elongation. Moreover, Gly35-1SNC preserved an intrinsically high antioxidant activity from reaming bioactive compounds of the seed and excellent biodegradability properties, as the films disintegrated in 48 h under lab-scale composting.

These findings suggest that avocado seed waste can be utilized to extract starch and develop films for active food packaging. The exploitation of avocado seeds, frequently treated as a byproduct, would contribute to the zero-waste and circular economy principles. However, more research is needed to enhance their mechanical and barrier qualities, as food packaging films typically demand sufficient stretchability and tensile strength to withstand external stress and retain their integrity, as well as high moisture and oxygen barrier properties. Solvent casting is a convenient laboratory-scale method for producing food packaging films, yet future research should focus on industrial applications and further improve the properties of the films by exploring extrusion and other conventional polymer-processing techniques such as blending.

## Figures and Tables

**Table 1:** Avocado-starch-based film compositions.

Film	Water (g)	ASS (g)	Glycerol (g)	SNCs (g)
Gly0	30	0.3	-	-
Gly15	30	0.3	0.045	-
Gly25	30	0.3	0.075	-
Gly35	30	0.3	0.105	-
Gly45	30	0.3	0.135	-
Gly35-1SNC	30	0.3	0.105	0.003
Gly35-3SNC	30	0.3	0.105	0.009
Gly35-5SNC	30	0.3	0.105	0.015

**Table 2:** Film thickness and luminance (L*) and color coordinates (a*b*) of avocado-starch-based films.

Sample	Thickness (µm)	L	a	b
Gly0	46.0 ± 2.0	84.62 ± 0.32	0.82 ± 0.34	21.38 ± 0.68
Gly15	53.0 ± 1.2	84.70 ± 0.76	1.04 ± 0.33	21.77 ± 1.83
Gly25	57.8 ± 0.4	84.38 ± 0.64	1.04 ± 0.21	21.29 ± 0.76
Gly35	59.3 ± 1.7	84.37 ± 0.66	1.08 ± 0.28	21.96 ± 1.42
Gly45	61.0 ± 4.0	84.98 ± 0.31	0.68 ± 0.22	21.44 ± 1.43
Gly35-1SNC	53.7 ± 3.7	83.70 ± 1.16	0.58 ± 0.26	20.06 ± 1.26
Gly35-3SNC	56.6 ± 1.0	83.37 ± 0.31	1.01 ± 0.19	20.46 ± 1.34
Gly35-5SNC	61.4 ± 4.7	82.80 ± 0.89	1.24 ± 0.13	21.01 ± 0.72

**Table 3:** Mechanical properties of avocado seed starch-based (ASS) films based on the effect of glycerol and starch nanocrystals (SNCs).

Film	Elastic Modulus (MPa)	Tensile Strength (MPa)	Elongation at Break (%)
(a) Mechanical properties of avocado seed starch (ASS)-based films based on the effect of glycerol.			
Gly15	1246.8 ± 172.8 ^a^	17.5 ± 1.1 ^a^	1.3 ± 0.7 ^a^
Gly25	420.1 ± 269.8 ^b^	4.8 ± 1.1 ^b^	15.0 ± 7.5 ^b^
Gly35	177.5 ± 42.7 ^c^	4.5 ± 0.3 ^b^	24.9 ± 1.7 ^c^
Gly45	109.3 ± 89.9 ^c^	3.7 ± 1.1 ^b^	22.5 ± 5.9 ^c^
F ratio	6.026	2.535	8.691
*p*-value	0.004 *	0.091 *	0.0005 *
(b) Mechanical properties of avocado seed starch (ASS)-based films based on the effect of starch nanocrystals (SNCs).			
Gly35	177.5 ± 42.7 ^a^	4.5 ± 0.3 ^a,b^	24.9 ± 1.7 ^a^
Gly35-1SNC	376.6 ± 126.9 ^b^	6.4 ± 1.4 ^a^	16.7 ± 1.7 ^b^
Gly35-3SNC	209.0 ± 113.6 ^a^	4.8 ± 1.4 ^a,b^	24.9 ± 4.2 ^a^
Gly35-5SNC	133.4 ± 69.8 ^a^	4.1 ± 1.3 ^b^	24.9 ± 3.8 ^a^
F ratio	1.264	4.014	2.97
*p*-value	0.312 *	0.021 *	0.065 *

Different letters in the column indicate significant differences according to Tukey’s test (*p* < 0.05). * Values are significant at *p* < 0.05.

**Table 4:** Barrier properties of avocado-starch-based films.

Reference	Gly0	Gly15	Gly25	Gly35	Gly45	Gly35-1SNC	Gly35-3SNC	Gly35-5SNC
WVP (g/mm.d.KPa)	-	5.1 ± 0.2	9.1 ± 1.7	13.5 ± 1.7	15.1 ± 0.8	8.8 ± 1.8	7.3 ± 1.2	10 ± 0.7
OTR(mL/m^2^ day)	>4000 *	>4000 *	>4000 *	11.0 ± 4.3	18.7 ± 2.6	9.8± 0.8	8.2 ± 3.0	12.2 ± 3.1

* Above the upper measurement range of the equipment for the tested area.

**Table 5:** Proposed compounds identified using GC-MS in avocado seed starch (ASS) and starch nanocrystals (SNCs), and starch-based films (Gly35).

tr (Min)	Proposed Compound	*m*/*z*	Molecular Formula
9.53	Avocadynofuran	248.10	C_17_H_26_O
10.01	(E)-Avocadynofuran	246.20	C_17_H_26_O
10.39	Avocadynofuran	244.18	C_17_H_24_O
11.29	n-Hexadecenoic acid	256.24	C_16_H_32_O_2_
11.55	2-(Pentadec-14-yn-1-yl)furan	274.23	C_19_H_30_O
11.59	(E)-2-(Pentadec-2-en-1-yl)furan	276.25	C_19_H_32_O
11.65	2-Pentadecylfuran	278.26	C_19_H_34_O
12.40	(E)-2-(Pentadeca-1,14-dien-1-yl)furan	274.23	C_19_H_30_O
12.46	(Z)-2-(Pentadec-1-en-1-yl)furan	276.25	C_19_H_32_O
13.18	9,12-Octadecadienoic acid (Z,Z)-	280.24	C_18_H_32_O_2_
13.50	2-((8Z,11Z)-Heptadeca-8,11-dien-1-yl)furan	302.26	C_21_H_34_O
14.22	2-((1E,8Z,11Z)-Heptadeca-1,8,11-trien-1-yl)furan	300.25	C_21_H_32_O
23.12	β-Sitosterol	414.39	C_29_H_50_O

## Data Availability

The raw data supporting the conclusions of this article will be made available by the authors on request.

## Supplementary Materials

The following supporting information can be downloaded at https://www.mdpi.com/article/10.3390/polym16202868/s1: Figure S1. FTIR spectra of avocado seed starch (ASS) and starch nanocrystals (SNCs). Figure S2. DSC of the avocado seed starch gelatinization. Figure S3. X-ray diffraction patterns of avocado-seed-starch-based films with different glycerol and SNC contents. Figure S4. Tensile properties of avocado-seed-starch-based films. Figure S5. Water vapor permeability of avocado-starch-based films.
