# Supplementary material for: Avocado Seed Starch-Based Films Reinforced with Starch Nanocrystals
Source: Polymers (Basel). 2024 Oct 10;16(20):2868. doi: 10.3390/polym16202868 (PMC11511395; doi:10.3390/polym16202868)
Supplement: Supplementary file 1 [file polymers-16-02868-s001.zip › polymers-3207050-supplementary.pdf]

polymers

MDPI

Article

Avocado Seed Starch-Based Films Reinforced with Starch Nanocrystals

Pedro Francisco Muñoz-Gimena [1], Alejandro Aragón-Gutiérrez [2], Enrique Blázquez-Blázquez [1], Marina Patricia Arrieta [3], Gema Rodríguez [1], Laura Peponi [1,*] and Daniel López [1,*]

1 Instituto de Ciencia y Tecnología de Polímeros (ICTP-CSIC), C/Juan de la Cierva 3, 28006 Madrid, Spain; pfmunoz@ictp.csic.es (P.F.M.-G.); enrique.blazquez@ictp.csic.es (E.B.-B.); gema@ictp.csic.es (G.R.)
2 Grupo de Tecnología de Materiales y Envases, Instituto Tecnológico del Embalaje, Transporte y Logística, ITENE, Unidad Asociada Al CSIC, C/Albert Einstein 1, Paterna, 46980 Valencia, Spain; alejandro.aragon@itene.com
3 Departamento de Ingeniería Química Industrial y del Medio Ambiente, Escuela Técnica Superior de Ingenieros Industriales, Universidad Politécnica de Madrid (ETSII-UPM), C/José Gutiérrez Abascal 2, 28006 Madrid, Spain; m.arrieta@upm.es
* Correspondence: lpeponi@ictp.csic.es (L.P.); daniel.l.g@csic.es (D.L.); Tel.: +34-915-622-900 (L.P. & D.L.)

Supplementary Information

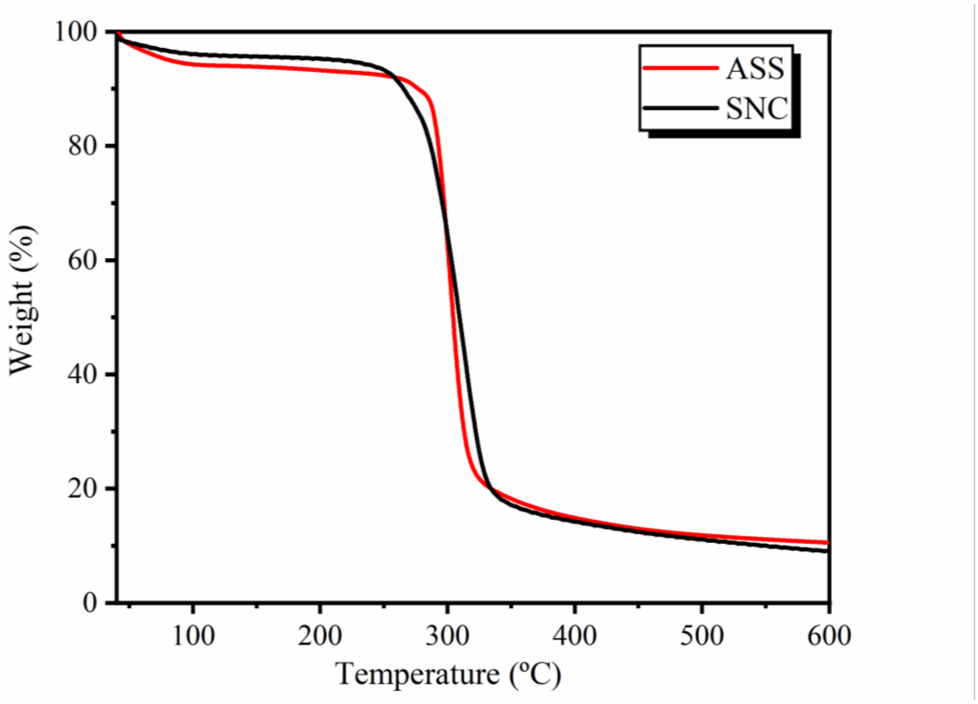

Figure S1. FTIR spectra of avocado seed starch (ASS) and starch nanocrystals (SNCs).

The wide band in the 3000–3600 cm^{-1} range corresponds to the stretching vibration hydroxyl (OH-) functional group. The vibration bands at 2923 cm^{-1}, 1,412 cm^{-1} and 1,242 cm^{-1} correspond to the carbon-hydrogen (C-H), carbon-carbon (C-C) and the deformation of the methyl group (C-H) bonds of the methyl groups respectively. The absorption bands at 1362 cm^{-1} and 1465 cm^{-1} are attributed to the plane O-H bending vibration in the glucose unit and 931 cm^{-1} to the O-H out-of-plane bending vibration. Other characteristic bands were observed at 1640 cm^{-1} indicating the presence of bonded water, and 1145, 1075 and

996 cm^{-1} were associated with the stretching vibration of the C-O bond, C-O-H and C-O-C groups in the anhydrous glucose ring, respectively.

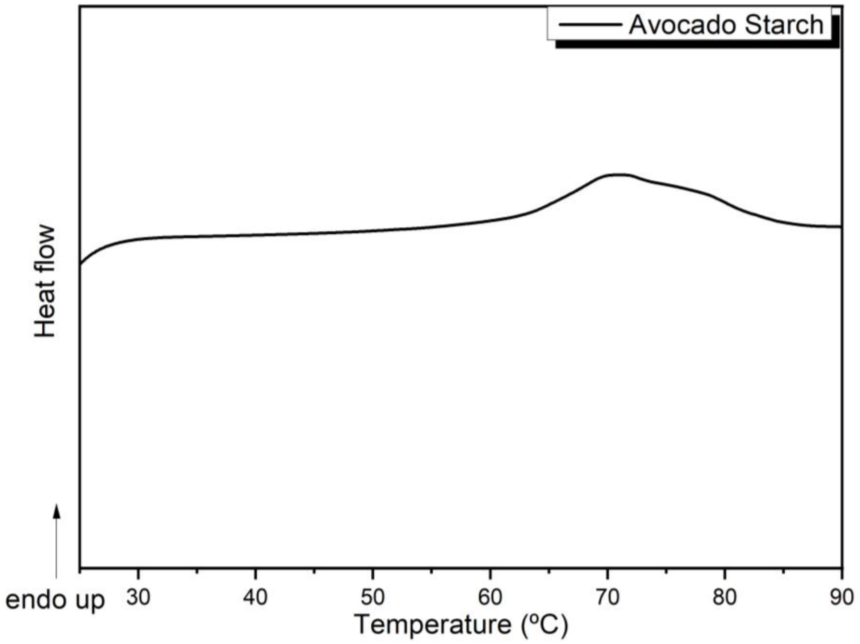

Figure S2. DSC of the avocado seed starch gelatinization.

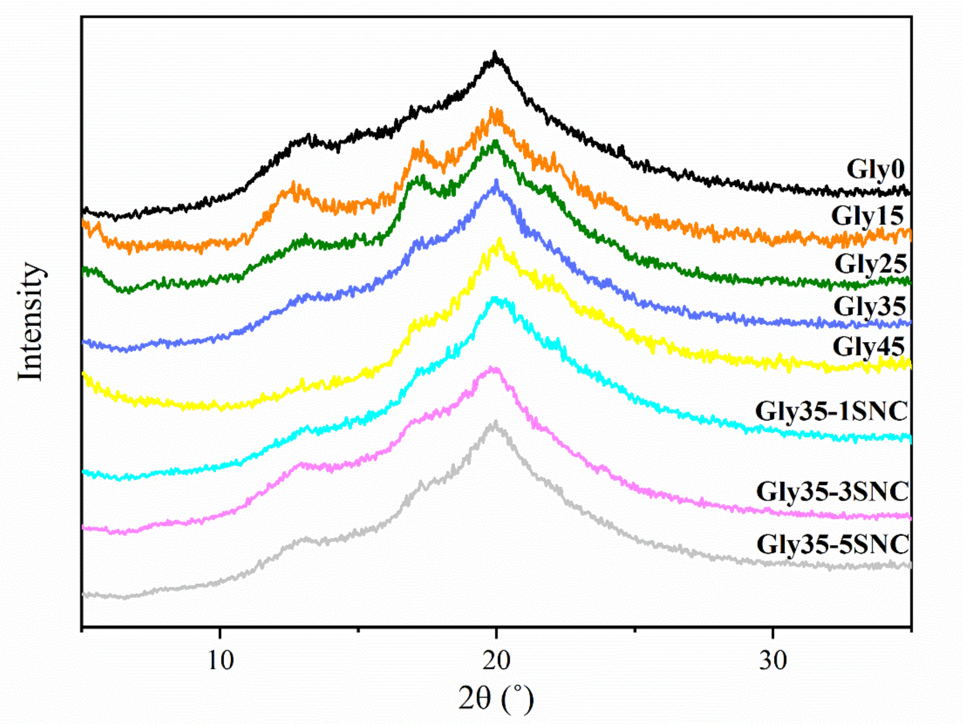

Figure S3. X-ray diffraction patterns of avocado seed starch-based films with different glycerol content and reinforced with starch nanocrystals.

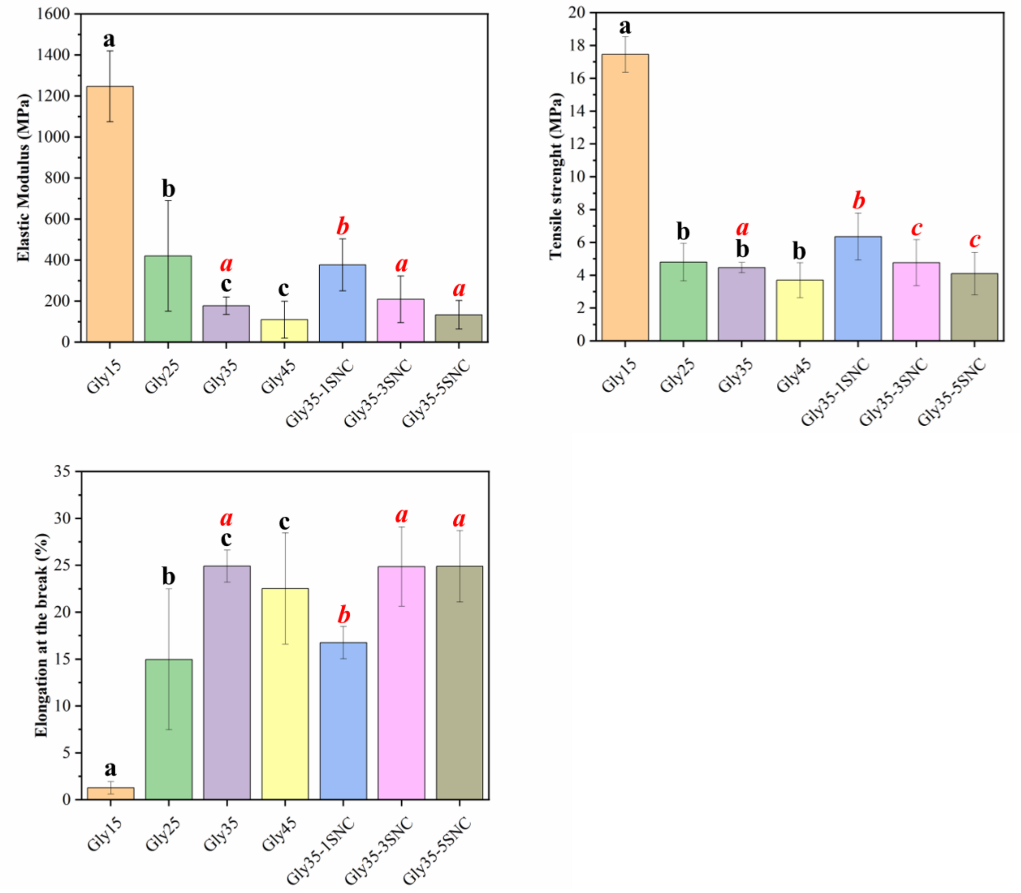

Figure S4. Tensile properties of avocado seed starch-based films: (a) Elastic Modulus, (b) Tensile strength, and (c) Elongation at the break. ANOVA analysis of the effect of glycerol (black) and SNC (red) concentration.

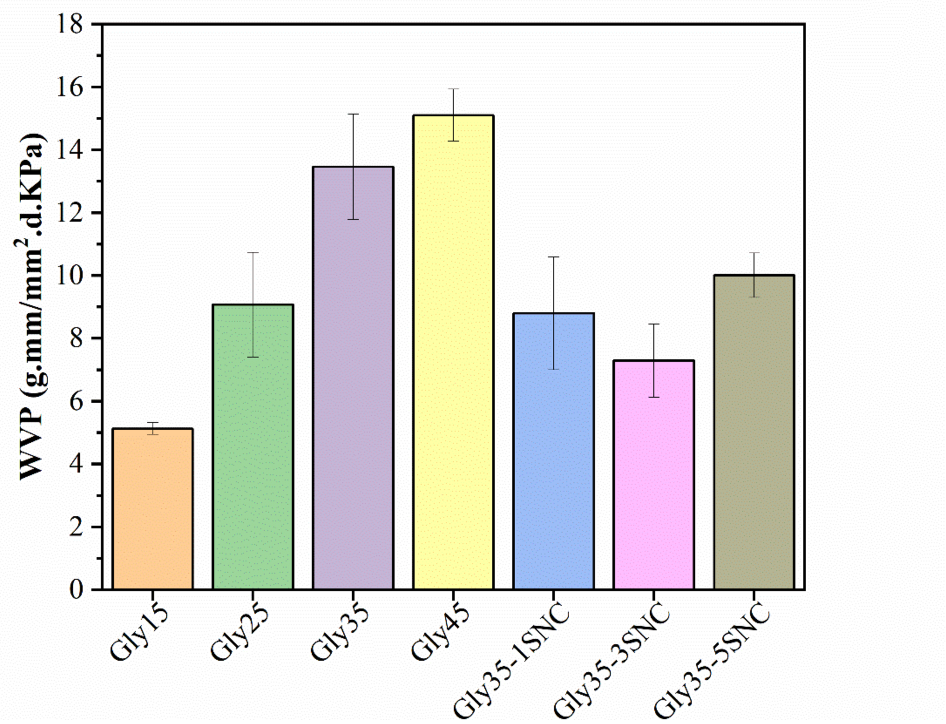

Figure S5. Water Vapor Permeability of avocado starch-based films.
